# Restricted participation: Drivers, experiences and implications of disability stigma in Ethiopia

**DOI:** 10.4102/ajod.v12i0.1085

**Published:** 2023-01-23

**Authors:** Esther Breffka, Caroline Jagoe, Susan P. Murphy, Belestie B. Tsegaw

**Affiliations:** 1 Department of Clinical Speech and Language Studies, Faculty of Arts, Humanities and Social Sciences, Trinity College Dublin, University of Dublin, Dublin, Ireland; 2 Department of Geography, Faculty of Science, Technology, Maths and Engineering, Trinity College Dublin, University of Dublin, Dublin, Ireland; 3 Department of Public Health, Faculty of Public Health, Health Care, Addis Continental Institute of Public Health, Addis Ababa, Ethiopia; 4 Department of Psychology, Faculty of Behavioral Science, Social Studies and Humanities, University of Gondar, Gondar, Ethiopia

**Keywords:** disability, disability stigma, community-based inclusive development, disability inclusion, social inclusion, epistemic injustice, disability rights, participation

## Abstract

**Background:**

Community-based inclusive development (CBID) acknowledges society’s critical role in supporting the active participation of persons with disabilities. However, research on how this approach relates to the context-sensitive socially situated barriers of disability stigma is underexplored.

**Objectives:**

This study aimed to understand the drivers and experiences of disability stigma in Ethiopia, from the perspective of persons with disabilities engaged in CBID programmes, and to establish how disability stigma acts as a barrier to participation.

**Methods:**

An inductive methodological approach guided the research design. Mixed methods were used including a narrative review of disabilities studies literature, 16 semi-structured interviews with persons with disabilities, and a quantitative survey of 970 persons with disabilities across three communities in Ethiopia.

**Results:**

Informed by theories of epistemic justice, this study identified specific indicators of meaningful participation and examined how these relate to experiences of disability stigma. The study found that the participation of adults with disabilities in society is restricted across different areas of life. Misconceptions about the causes of disability and social perceptions regarding the capacities of persons with disabilities are found to exacerbate stigma and act as a barrier to participation.

**Conclusion:**

Targeted efforts to challenge internalised norms and harmful beliefs within CBID approaches are required to address disadvantages arising from embedded disability stigma.

**Contribution:**

This study makes conceptual, empirical and practical contributions that advance insights into the relationship between disability stigma and participation in Ethiopia and the dimensions of epistemic justice relevant to understanding the nature and drivers of disability stigma.

## Introduction

The United Nations (UN) Convention on the Rights of Persons with Disabilities (CRPD) (2006) identifies the full and effective participation of persons with disabilities as one of its grounding principles. Community-based inclusive development (CBID), with its roots in community-based rehabilitation, seeks to operationalise this principle through the model of a human rights-based approach. CBID adopts a holistic approach, activating the role of communities to reduce barriers that restrict full participation (CBM International 2020; World Health Organization [WHO] et al. 2010:4). Most research on CBID has focused on the interrelatedness of domains such as health, inclusive education and livelihoods. Although disability stigma has emerged as a core barrier to the full participation of persons with disabilities in society (Mostert 2016; Rohwerder 2018), research on the role and effect of stigma in the context of CBID has been limited, and research on CBID, in general, is lacking (White, Saran & Kuper 2018).

Considering that CBID aims at building inclusive communities (CBM International 2020; WHO et al. 2010:4ff), there is an urgent need to better understand the experiences and effects of disability stigma on the participation of persons with disabilities in community life. Further questions of whether disability stigma creates a form of social powerlessness that systematically disadvantages persons with disabilities in their opportunities to meaningfully participate in society are also underexplored.

Previous studies have investigated the relationship between disability, stigma and disability rights; however, their focus is based on assumptions around the existence of disability stigma rather than critically interrogating the drivers and experiences associated with this as experienced by persons with disabilities (Grischow et al. 2018; Mostert 2016; Rohwerder 2018). Other disability-related literature targets predominantly health and education (Mostert 2016; White et al. 2018:21ff.), and interventions for specific disabilities (cf. Hartog et al. 2020; Heijnders & Van der Meij 2006; Saran, White & Kuper 2019). Furthermore, it is widely recognised that disability is primarily analysed from a medical rather than from a human rights perspective, meaning that topics such as advocacy, empowerment, participation and social inclusion are underrepresented in the existing literature (White et al. 2018:21ff.). Against this backdrop, this research aims to gain a better understanding of the experiences of persons with disabilities with regard to disability stigma and the implications for their participation in society.

Following the application of the social model that regards disability as socially constructed, this research is based upon the understanding of disability stigma as laid out by Goffman (1963:13ff.) and utilises the nuanced framework by Link and Phelan (2001) that integrates the dynamics of societal power structures as essential components of stigma. Stigma here is defined as ‘[*t*]he co-occurrence of its components – labelling stereotyping, separation, status loss, and discrimination – and further indicate that for stigmatisation to occur, power must be exercised’ (Link & Phelan 2001:363).

To analyse the relationship between disability stigma and participation, the literature commonly refers to four different types of stigma, depending on the actors involved in the process of stigmatisation: ‘felt stigma’ refers to the fear of stigmatisation (individual level) (Brown, Macintyre & Trujillo 2013:50), ‘enacted stigma’ as the act of discrimination (directed interaction among individuals with and without disability) (Brown et al. 2013), ‘public stigma’ as the (re)production of negative attitudes (societal level) (Amoah 2016:42) and self-stigma as the process of internalising, accepting and identifying oneself with these negative associations (feedback from society to individual) (Brown et al. 2013).

Recognising the nature of unequal relations of power and knowledge (Kidd, Medina & Pohlhaus 2017:303), this research engages with theories of epistemic injustice to understand how these relations influence participation. First articulated in the work of Miranda Fricker, epistemic justice entails two specific types of harm that can be experienced by a person (Fricker 2006) or group (Anderson 2012), in their capacity as a knower – testimonial and hermeneutical injustice. According to Fricker (2007:1), ‘testimonial injustice occurs when prejudice causes a hearer to give a deflated level of credibility to the speaker’s. This form of injustice carries implications for those seeking meaningful participation in community life – that their voice and perspectives be recognised as credible and listened to:

[*H*]ermeneutical injustice occurs at a prior stage when a gap in collective interpretive resources puts someone [or group] at an unfair disadvantage when it comes to making sense of their social experience. (Fricker 2007:1)

This form of injustice gives us a window into understanding the dynamics of disability stigma, how this is embedded in cultural practice, histories and norms, and places persons with disabilities at an unfair disadvantage from the outset. The power to make sense of and give meaning to one’s own social experience strongly relates to one’s position in society. It is influenced by the distribution of power and knowledge in society (Fricker 2006). Full participation in social institutions (Anderson 2012), then, would require the recognition of the value and credibility of the testimony of persons with disabilities and also an active role for persons with disabilities in shaping understandings of their own social experiences, rather than having these understandings shaped by pre-existing social prejudice or other dominant groups.

Given that persons with disabilities are often characterised as being socially powerless (Nepveux & Beitiks 2010), this study examines whether the case of disability stigma can be described as a form of systematic hermeneutical injustice, whereby systematic hermeneutical injustice – different from incidental hermeneutical injustice – originates from ‘a structural prejudice in hermeneutical resources’ (Fricker 2006:100). Thus, the use of systematic hermeneutical injustice can be described as a tool to explain the nature of persons with disabilities’ exclusion through linking disability stigma and assumed capacity to participate. This helps us to explain why even in circumstances where participation takes place, persons with disabilities can find that their voices are not heard, and their testimony is not given sufficient consideration and weight. Based on this theoretical foundation, this research applies the concept of systematic hermeneutical injustice from its origin within feminist epistemology (Giladi & McMillan 2018:1) to another dimension of marginalisation disability. Hence, this study critically interrogates how the participation of persons with disabilities in society is influenced by pre-existing, socially embedded unequal relations of power and knowledge.

The study seeks to answer the following question: how does disability stigma affect the participation of persons with disabilities? We examine this through a case study of the experiences of persons with disabilities in the South Gondar Zone of Ethiopia. We begin with a narrative review of the literature on disability stigma to explore what is known about the underlying drivers and experiences of disability stigma. We then share results and insights from a series of interviews and survey data gathered with and from persons with disabilities to explore how disability stigma is perceived in Ethiopia and how this affects active participation in society. The study makes an empirical contribution to knowledge regarding the specific insights and experiences of disability stigma and barriers to participation of persons with disabilities in Ethiopia. The practical and theoretical implications of this contribution are also explored.

## Research methods and design

This is a transdisciplinary study engaging academic researchers with an international disabilities organisation (IDO), locally based disabled persons’ organisations (DPOs), and persons with disabilities engaging with these agencies. The results shared here are part of a wider study designed to identify opportunities for improvement of CBID programmes in Ethiopia. Based on an inductive approach, the research design and data collection unfolded over three phases, beginning with project meetings in Ethiopia in 2019. Here, the overarching design of the research was developed, and key areas of focus agreed. The second stage of the research entailed a survey of persons with disabilities across three communities in the South Gondar Zone of Ethiopia, namely Debre Tabor, Dera and Wereta. All communities are actively engaged in CBID projects implemented through local DPOs. The areas are characterised by a relatively high level of poverty and weak infrastructure, are largely rural communities engaged in subsistence agriculture, with traditional social systems, and ethnic belonging to the Amhara. The third stage entailed qualitative data collection through semi-structured interviews, with members of the community with disability, specifically on experiences of disability stigma and participation.

Although disruptive, the onset of coronavirus disease 2019 (COVID-19) presented an opportunity to more actively engage DPOs and persons with disabilities in the data collection processes. Local enumerators, all of whom self-identified as persons with disabilities, where trained in data collection techniques and were the leaders in collecting the insights from within their own communities.

### Data collection

#### Narrative review of the literature

To systematically select the most relevant literature, a five-stage process was applied. Firstly, identifying literature via the use of research engines and citation tracking; secondly, applying accessibility criteria; thirdly, categorising the literature to assess its eligibility; fourthly, screening the literature to exclude non-topic specific literature; and fifthly, critical appraisal to ensure the literature’s trustworthiness (Virendrakumar et al. 2018). In keeping with a structured approach to literature synthesis and to maintain transparent links between the synthesis data and the synthesis reported (Campbell et al. 2019), the data sources are cited in the analysis that follows. A full list of data sources used in the narrative synthesis is listed in Online Appendix 1, and those cited in the text also appear in the reference list.

For the identification of primarily academic literature, the following search engines were used: APA PsycArticles, PubMed, Science Direct, The host University’s Library Search function (Stella Search), and Web of Science. Google Scholar was used to extend the scope to non-primarily academic articles, including grey literature, policies and legal documents. Policies and legal documents were further manually retrieved from the Office of the United Nations High Commissioner for Human Rights’ Anti-discrimination Library, given the lack of a precise keyword search option. Documents were accessed by using the keyword function: (‘disability’ AND ‘Ethiopia’ AND ‘stigma’ AND [‘rights’ OR ‘social inclusion’ OR ‘social exclusion’ OR ‘participation’]). This choice was reasoned by the study’s focus on disability-related stigma. The inclusion of grey literature and legal materials was necessary to gather insights and to balance the lack of academic research on this topic in low-income locations. Literature was not limited by data of publication.

#### Primary data collection

The second component of the study involved a survey across the three communities. The survey was administered between December 2019 and January 2020 using purposive sampling, with a maximum variation sample in terms of disability, gender and geography. All participants were over the age of 18. The data collection tools were developed with reference to the Washington Group Questions (short set; WGQ-SS), the CBM Monitoring of Inclusion, the University of Sydney and the Cheshire Foundation Action for Inclusion Bahir-Dar Project Office Survey Tool. Administration of the questionnaire with in-depth interviews was conducted with 970 adults with disabilities (509 men and 461 women). Types of disability were grouped by functional difficulties based on the adapted WGQ-SS and classified into seven categories (Table 1).

**TABLE 1:** Survey participants.

Domain of functional difficulty	Men		Women		Total	
	*N*	%	*N*	%	*N*	%
Vision	153	30.1	153	33.2	306	31.5
Hearing	41	8.1	50	10.8	91	9.4
Cognitive	29	5.7	24	5.2	53	5.5
Mobility	260	51.1	205	44.5	465	47.9
Multiple	16	3.1	18	3.9	34	3.5
Other	7	1.4	10	2.2	17	1.8
No response	3	0.6	1	0.2	4	0.4

**Total**	**509**	**100.0**	**461**	**100.0**	**970**	**100.0**

The third stage of the study involved qualitative data collection through semi-structured interviews with 16 adults with disabilities in the respective settings. These took place between May and July 2021. Participants were selected in collaboration with local DPOs. The structure of the interview and the choice of questions were guided by the ‘funnel’ principle, moving from broad and open to more critical and narrow questions, including content-mapping and content-mining questions as well as in-depth probing. Following the literature review and research question, a topic guide and a semi-structured interview guide on participation were created and discussed with the co-researchers. An accessible version of the WGQ-SS was compiled in the local language for the purpose of disability description. In close consultation with the practitioner organisations, four persons with disabilities were chosen as co-researchers based on the following requirements: lived experience of disability, DPO membership, and sensitivity around disability and gender issues. All co-researchers were adults in the age range of 18–50 years old.

Interviews were conducted in the local language, Amharic, and translated into English for analysis.

### Data analysis

This narrative review critically examined ‘how and why incidents are storied, not simply the content to which language refers’ (Riessman 2008:11). The use of content analysis allows for systematic framing of existing models, focusing on extracting the main drivers of disability stigma and types of participation restrictions. Thematic coding was utilised to identify themes and categories across the literature by identifying the main themes, recognising subsequent themes, sub-grouping the literature and identifying the main concepts.

Descriptive analysis of survey data using a subset of questions was used to explore if and to what extent disability stigma is perceived as restricting participation in society for persons with disabilities. Narrative analysis of the qualitative data was used to explore the individual experiences of persons with disabilities around participation. The data were coded and thematically grouped using Microsoft Word and Excel.

Coding from the International Classification of Functioning, Disability, and Health (ICF; WHO 2017) was used as an analytic tool, which can support the human rights approach to disability as it incorporates impairment and environment to assess disability. Within the ICF participation is measured around multiple domains. For the analysis of this study, the authors focused on the domains of self-care; domestic life; interpersonal interactions and relationships; major life areas; and community, social and civic life (ICF chapters p5–p9). Analysis was based on the frequency of participation and contextual factors identified in the literature, meaning that values do not indicate the extent of influence, but rather the frequency of reporting within the reviewed data.

### Ethical considerations

Ethical approval was granted from Ethics School of Natural Sciences (SNS) Research Ethics Policy School of Natural Sciences, Trinity College Dublin.

The Institutional Review Board at a large Irish university approved the research (Online Appendix 1). Data collection was undertaken with respect to the common principles of ethical research, and enumerators were specifically trained for the purpose of this study given that this research engages with participants at risk of vulnerability. Research participants were informed about all components of the project and their right to privacy, anonymity and access to data. Enumerators explained that participation was voluntary and that participants had the right to withdraw and ask for further explanations at any stage of the research. When participants were informed and consented to participation – verbally or written – and their safeguarding ensured, the interview process was undertaken.

The sensitive nature of this research poses limitations that require consideration. This study engages with perceptions and social constructions, which require reflection on power differentials, particularly concerning disability, socio-economic background and historical racial inequalities that can influence the research process. The researchers are aware of their positionality as international expert practitioners and researchers, collaborating with locally based practitioners and communities. Consequently, significant efforts to instil critical reflexivity into the research process have been made.

**TABLE 2:** Interview participants.

Participant ID	District†	Gender‡	Age	Domain of functional difficulty§	Participation in DPO meetings	Participation in Kebele meetings
P01	DT	M	20–29	Seeing^3^	Yes	Yes
P02	DT	W	30–39	Seeing^2^; Walking^2^	Yes	Yes
P03¶	D	W	30–39	-	No	No
P04	W	W	20–29	Walking^3^	Yes	Yes
P05	DT	W	40–49	Seeing^4^; Walking^2^	Yes	Yes
P06	DT	M	30–39	Walking^2^	Yes	Yes
P07††	DT	W	20–29	Seeing^4^	Yes	Yes
P08††	DT	M	30–39	Seeing^4^; Self-care^2^	Yes	Yes
P09	D	W	20–29	Walking^3^; Self-care^3^	Yes	No
P10	D	M	30–39	Walking^2^	Yes	No
P11	W	M	20–29	Seeing^4^; Walking^2^; Cognition^2^; Communication^2^	Yes	Yes
P12††	W	M	20–29	Walking^2^	Yes	Yes
P13*	W	W	20–29	Seeing^4^	No	No
P14*	DT	M	30–39	Walking^2^	Yes	Yes
P15*	DT	W	30–39	Hearing^2^; Walking^4^	Yes	Yes
P16*	D	M	20–29	Walking^2^	Yes	No

† , Abbreviations indicates district level, DT-Debre Tabor, D-Dera, W-Wereta;

‡ , Abbreviations indicates response category, M-Man, W-Woman;

§ , Self-identified as persons with disabilities with domain of difficulty specified on WGQ-SS. Superscripts indicate response category 1-no difficulty, 2-some difficulty, 3-a lot of difficulty, 4-cannot do at all. Participants self-identified as persons with disabilities. Therefore, a threshold of disability was not prescribed, but the WGQ-SS used to characterise the functional difficulties experienced. The signs ‘¶‘ and ‘††’ are utilized as codes in the narrative analysis to transparently display responses:

¶ -participants self-identified as a person with a disability, but the WGQ-SS were not sensitive to the nature of their difficulties;

†† -participants not (clearly) self-identified, but all self-identified as persons with disabilities in the interviews.

* , Asteria indicate that the data was collected through the piloting.

To minimise power imbalances and encourage persons with disabilities in their capacity as knowers, this research strives to focus on narratives from persons with disabilities themselves. A key challenge for the research team was to avoid the reproduction of disability stigma through the research process. To minimise the extent to which this research replicates the widely accepted recognition of persons with disabilities as particularly marginalised and vulnerable, it is set in an emancipatory frame (Barnes 2009:461ff.), meaning that the perspectives and knowledge of persons with disabilities shaped the research design and outcomes. It actively recognised and respected all participants as active knowers and those best positioned to make sense of their own social experiences. This research sought to counter what Nepveux and Beitiks (2010) refer to as the tendency of Western neo-colonial narratives to depict African persons with disabilities as inferior. To decolonise the research process (Ndimande 2018), research participants were interviewed by local researchers in the local language and in close consultation with local practitioner organisations, including local DPOs.

## Results

### Narrative synthesis of literature

**FIGURE 1:**
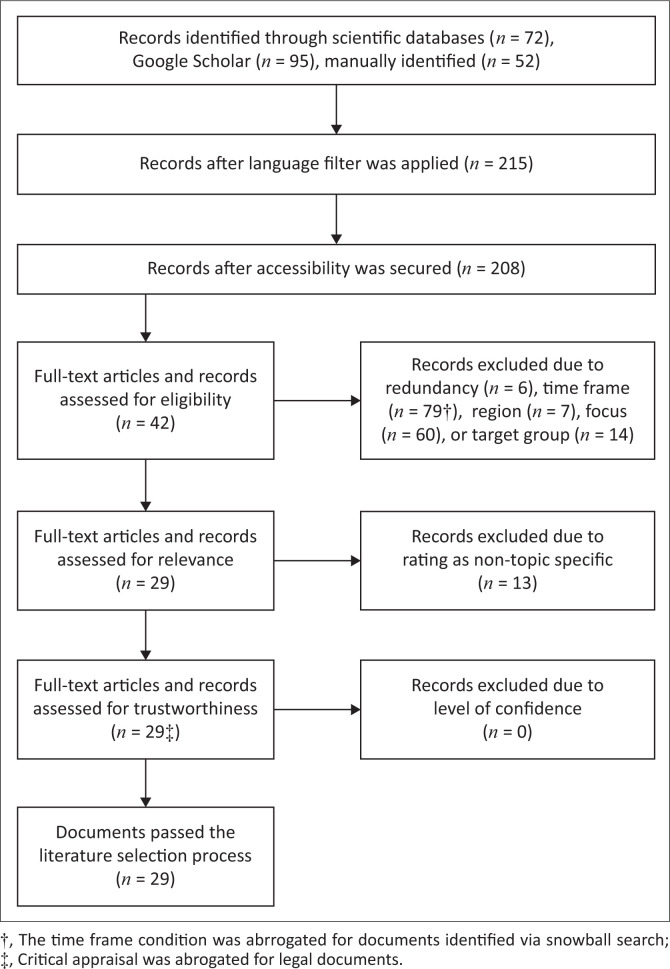
Flow diagram of the literature selection process.

After the initial search of academic databases and grey literature, 219 documents were retrieved. Through an application of the inclusion criteria, 29 texts were selected for review (Figure 1).

The 29 selected documents included 20 academic research articles, grey literature sources and three legal documents. Almost half of the documents can be attributed to the social sciences, with the remaining split between a human rights-based approach and the medical approach.

Initial review of the articles indicates that recognising disability stigma as a barrier to persons with disabilities’ legal rights entitlements or to the severity of disability remains under-researched. Most of the articles focus on experiences around disability stigma as social stigma, with a focus on disability in relation to persons with mental disorders. The focus of disability type may be rooted in the widely accepted view that persons with mental disabilities are especially vulnerable to disability-related stigma (Rohwerder 2019a).

Five umbrella factors can be identified from the literature (Table 3), which appear to be non-exclusive and relational. Lacking awareness describes missing knowledge and interest about disability. Misconception about the causes encompasses misdeed of persons with disabilities, misdeed of ancestors, supernatural forces, for example, evil spirit or witchcraft, punishment from God or curse of God, and other causes. Fear of negative impact when contact with person with disability defines a person’s negative attitude or discriminatory behaviour against persons with disabilities because of being afraid of negative implications, for example, status loss resulting from contact with persons with disabilities. Assumptions about persons with disabilities include disbeliefs about persons with disabilities’ ability, behaviour and nature. Discriminatory policies refer to legislation that contributes to unequal treatment of persons with disabilities compared with persons without disabilities and derogatory language used within. Within the literature reviewed, the highest explanatory power (in terms of the highest frequency of reporting) is attributed to a lack of awareness and misconceptions about the causes of disability. Underlying traditional beliefs and social norms were most commonly cited as origin of misconceptions, predominantly the assumption that supernatural forces or punishment from God led to an impairment. However, not all traditional beliefs result in negative perceptions of disability (cf. Mostert 2016:9).

**TABLE 3:** Summary of the literature.

Source	Lacking awareness	Misconception about the causes	Fear of negative impact when contact with persons with disabilities	Assumptions about persons with disabilities	Discriminatory policies
Abah 2017	-	-	-	-	-
Amoah 2016	✓	✓	✓	✓	-
Arulanantham 2014	✓	✓	-	-	-
Ebuenyi et al. 2018	✓	✓	✓	✓	✓
Ebuenyi et al. 2019	-	-	-	✓	✓
Habtamu et al. 2015	-	-	-	✓	-
Habtamu et al. 2018	-	-	-	-	-
Lord & Stein 2013	-	-	-	-	✓
Mfaofo-M’Carthy & Grishow 2017	✓	✓	✓	✓	-
Mostert 2016	✓	✓	✓	✓	✓
Rohwerder 2018	✓	✓	-	✓	✓
Rohwerder 2019a	✓	✓	✓	✓	-
Rohwerder 2019b	✓	✓	-	✓	✓
Rohwerder 2020	✓	✓	-	✓	-
Shahvisi et al. 2018	✓	✓	-	✓	-
Spittel et al. 2019	✓	✓	✓	✓	✓
Stangl et al. 2019	✓	✓	✓	✓	✓
Surur et al. 2017	-	✓	✓	✓	-
Tesfaw et al. 2020	✓	✓	-	-	-
Tesfaye et al. 2020	-	-	-	-	-
The Advocates for Human Rights 2016	-	-	-	-	✓
Tirfessa et al. 2019	-	-	-	-	-
Tora et al. 2018	✓	✓	-	✓	-
Tsegay et al. 2018	✓	✓	-	✓	✓
UN CRPD Committee 2016	-		-	-	-
UNDESA 2016	✓	✓	✓	✓	-
Van Brakel 2006	✓	✓	-	-	-
Van’t Noordende et al. 2020	-	-	-	-	-
Virendrakumar et al. 2018	✓	✓	-	-	✓

While the literature acknowledges social norms and traditional beliefs as general driver of disability stigma, less effort has been made to disaggregate the data and to derive disability-specific patterns (Grischow et al. 2018; Mostert 2016; Rohwerder 2018). Public stigma of persons with disabilities, for example, results from different beliefs. Persons with mental disorders are often misassociated with dangerous or unpredictable behaviour (Ebuenyi et al. 2018; Habtamu, Alem & Hanlon 2015; Mfaofo-M’Carthy & Grishow 2017; Mostert 2016; Rohwerder 2019a, 2019b; Spittel, Maier & Kraus 2019; Stangl et al. 2019; Surur et al. 2017), whereas persons affected by leprosy are perceived as not hygienic and infectious (Amoah 2016; Rohwerder 2018; Stangl et al. 2019).

### Contextual factors: Facilitators and barriers

Following from the identification of key themes within literature, the ICF was utilised to translate these into environmental and personal factors to analyse more systematically what type of factors appear to drive disability stigma. Table 4 details which of the ICF environmental chapters were predominant within the reviewed literature by health condition (ICD-11; WHO 2019). Findings from the literature support the assumption that attitudes are a major concern in the lived experience of persons with disabilities. Four (e1, e3, e4 and e5) of the five environmental chapters are explicitly stated as barriers to the participation of persons with disabilities.

**TABLE 4:** Identification of environmental factors (International Classification of Functioning, Disability, and Health chapters) within reviewed literature.

Health condition (ICD-11 code) reported in literature	Number of records reporting on health condition	Number of mentions of ICF environmental factors† (in relation to no. of records reporting on health condition‡)				
		e1	e3	e4	e5	Σ
Disability, unspecified (X)	12	5/12	3/12	12/12	11/12	**31**
Leprosy, unspecified (1B20.Z)	5	0/5	0/5	5/5	5/5	**10**
Lymphoedema due to podoconiosis (BD93.14)	4	0/4	0/4	4/4	4/4	**8**
Mental, behavioural or neuro-developmental disorders (06)	4	0/4	0/4	4/4	3/4	**7**
Disorder of intellectual development, severe (6A00.2)	3	0/3	1/3	3/3	3/	**7**
Schizophrenia, unspecified (6A20.Z)	2	0/2	1/2	2/2	2/2	**5**
Human immunodeficiency virus disease without mention of associated disease or condition, clinical state unspecified (1C62.Z)	2	0/2	0/2	2/2	1/2	**3**
Mood disorders, unspecified (6A8Z)	1	0/1	1/1	1/1	1/1	**3**
Mental, behavioural or neurodevelopmental disorders, unspecified (6E8Z)	1	0/1	1/1	1/1	1/1	**3**
Lymphatic filariasis (1F66.3)	1	0/1	0/1	1/1	1/1	**2**
Unspecified malignant neoplasms of ill-defined or unspecified sites (2D4Z)	1	0/1	0/1	1/1	1/1	**2**
Obesity, unspecified (5B81.Z)	1	0/1	0/1	1/1	1/1	**2**
Epilepsy or seizures, unspecified (8A6Z)	1	0/1	0/1	1/1	1/1	**2**
Albinism or other specified genetically-determined hypomelanotic disorders (EC23.2)	1	0/1	0/1	1/1	1/1	**2**
Dementia, unknown or unspecified cause (6D8Z)	1	0/1	0/1	1/1	0/1	**1**
Total number of mentions of ICF environmental chapters within total no. of records§	**40**	**5/40**	**7/40**	**40/40**	**36/40**	**88**
Percentage (%) of mentions of ICF environmental chapters within total no. of records§	**-**	**13 %**	**17.5 %**	**100 %**	**90 %**	-

† , Acronym indicates identified ICF environmental factors (chapters), e1-Products and technology, e3-Support and relationships e4-Attitudes, e5-Services, systems and policies;

‡ , Information utilized for this figure is not data-driven as the literature reviewed is not primarily quantitative. It rather displays the ICF environmental factors (chapters) that have received attention within the literature, and were most frequently reported;

§ , The total number of records (40) differs from the total number of documents (29) included in the literature review as some documents reported on more than one health condition. This was considered in the analysis by using the total no. of records (40) as baseline.

Although products and technology (e1) are mentioned as a barrier, the focus in the documents reviewed is rather on the social aspects of disability. The counts allocated in support and relationships (e3) highlight that lacking support from the immediate family, community and people in authority negatively affect persons with disabilities and are often associated with higher internalised stigma.

The reviewed literature provides clear evidence of social attitudes as barrier to and/or facilitator of participation. Persons with disabilities experience negative attitudes at home in the immediate family circle, within their community, at work and from service providers. It is widely found that social norms and beliefs are particularly problematic and prevent participation (Rohwerder 2019a, 2019b).

Services, systems and policies (e5) are also mentioned as negatively influencing persons with disabilities’ daily lives. This is particularly highlighted in terms of civil protection services, systems and policies, legal services, health services, systems and policies, education and training services, systems and policies, and labour and employment services, systems and policies.

As a complement to the recognition of environmental factors, the ICF incorporates personal factors as facilitators or barriers to a person’s functioning. Yet, its operationalisation remains challenged by the absence of an exhaustive list, which often leads to siloed considerations of the personal and environmental dimensions. Here, the authors try to dissolve those siloes by firstly identifying (a non-exhaustive list of) personal factors mentioned in the reviewed literature as barriers (negative value) or facilitators (positive value) to persons with disabilities’ participation (Table 5) and secondly acknowledging overlaps between the personal and environmental factors.

**TABLE 5:** Identified personal themes and factors to persons with disabilities’ participation within reviewed literature.

Personal themes and factors identified within reviewed literature	Number of records reporting on personal theme (in relation to total no. of records†)	Number of mentions of personal factors (facilitators/barriers‡) (in relation to total no. of records per theme§)	
		Facilitators	Barriers
**I Psychological processes of meaning-making**	**37/40**	**2/37**	**35/37**
1 Self-stigma (high)	-	-	−19/37
2 Self-esteem (low)	-	-	−12/37
3 Perceived stigma	-	-	−4/37
4 Self-esteem (high)	-	1/37	-
5 Motivation	-	1/37	-
**II Poor socio-economic status due to limited access to material resources, such as:**	**21/40**	**-**	**21/21**
1 Poverty	-	-	−5/21
2 Education (low)	-	-	−5/21
3 Unemployment	-	-	−4/21
4 Socio-economic status (poor)	-	-	−4/21
5 Income (low)	-	-	−2/21
6 Occupational prestige (low)	-	-	−1/21
**III Poor socio-cultural status due to underlying socially and culturally constructed norms around/in:**	**19/40**	-	**- 19/19**
1 Gender (being a woman)	-	-	−9/19
2 Marital status (not being married)	-	-	−4/19
3 Rural living areas	-	-	−3/19
4 Ethnicity	-	-	−2/19
5 Sexual orientation	-	-	−1/19
**IV Domain and level of functional difficulty;** **treatment-related factors**	**16/40**	**-**	**16/16**
1 Level of functional impairment (high)	-	-	−5/16
2 Domain of functional impairment (mental)	-	-	−4/16
3 Age (old)	-	-	−3/16
4 Actual level of functioning	-	-	−2/16
5 Medication side effects	-	-	−2/16

† , The total number of records (40) differs from the total number of documents (29) included in the literature review as some documents reported on more than one health condition. This was considered in the analysis by using the total no. of records (40) as baseline;

‡ , After the identification of key themes and subgrouping, a personal factor was assigned -1 or +1, depending on the following condition: if a source or responded acknowledged one of the identified personal factors as a barrier to persons with disabilities’ participation, it was coded - 1, if it was labelled as a facilitator, +1. Accordingly, values do not indicate the extent of influence, rather the frequency of reporting within the reviewed data;

§ , Information utilized for this figure is not data-driven as the literature reviewed is not primarily quantitative. It rather displays the personal factors that have received attention within the literature, and were most frequently reported.

Within the reviewed literature, most explanatory power is attributed to the overarching theme of psychological processes of meaning-making, suggesting that processes of internalising stigma manifest and multiply the already existing effect of persons with disabilities’ social exclusion, as the excluding effect is compounded with a self-isolating effect (Habtamu et al. 2015; Mostert 2016; Rohwerder 2020; Tsegay et al. 2018). The distribution of personal factors gives further account to intersectionality in the context of disability, negatively affecting women with disabilities, as well as the interrelatedness between limited access to resources because of non-inclusive societal structures and systems and actual participation opportunities (Arulanantham 2014; Habtamu et al. 2015; Mostert 2016; Rohwerder 2018, 2020; Tsegay et al. 2018; Van‘t Noordende, Aycheh & Schippers 2020).

### Experiences of disability stigma: Restricted participation

The literature pointed to clear patterns suggesting that there are experiences of restrictions that are unique to persons with disabilities, that is, that are not experienced by their peers without disabilities (Abah 2017). Despite this recognition, knowledge regarding the extent and type of participation domain affected is still lacking (Rohwerder 2018; White et al. 2018). Henceforth, this section strives to address the identified knowledge gap by operationalising participation according to the ICF.

Allocating the key themes from the narrative analysis to the ICF participation chapters (p5–p9) supports the hypothesis that persons with disabilities are restricted in all areas of their life (Rohwerder 2020; Tora et al. 2018; UN Committee on the Rights of Persons with Disabilities [UN CRPD Committee] 2016; United Nations Department of Economic and Social Affairs [UNDESA] 2016). Nevertheless, not all areas are similarly affected. Restrictions related to community, social and civic life (p9) and interpersonal interactions and relationships (p7) were most frequently recorded, followed by major life areas (p8), self-care (p5) and domestic life (p6).

Disaggregating the data to the second level throws light on the distribution within each chapter and enables us to derive patterns regarding the relationship between the type of impairment and participation restriction. Restrictions related to self-care were dominated by limitations in looking after one’s health. Several studies indicate a relationship between felt stigma and internalised stigma with non-adherence of medication or treatment because of persons with disabilities’ fear that taking medication would make their impairments visible and result in social exclusion. This association was validated for different types of impairments, for example, mental disorders and podoconiosis (Amoah 2016; Stangl et al. 2019; Surur et al. 2017; Tesfaw, Kibru & Ayano 2020; Tora et al. 2018; Van Brakel 2006), indicating that internalised stigma seems to be a characteristic of different types of impairments.

For domestic life, acquiring a place to live and acquisition of goods and services were particularly visible in the literature and associated with three arguments: the perception of persons with disabilities as not capable of living independently; lacking financial resources; and no permission by the family to live independently (Abah 2017; Amoah 2016; Arulanantham 2014; Stangl et al. 2019; Tesfaw et al. 2020; Tirfessa et al. 2019; Virendrakumar et al. 2018).

The counts associated with interpersonal interaction and relationships (p7) are more distributed across the literature, but predominantly attributed to informal social and intimate relationships. Social stigma was often linked to misconceptions about persons with disabilities and their capacity to marry (Amoah 2016; Habtamu et al. 2015; Rohwerder 2019a, 2019b; Tora et al. 2018; Van Brakel 2006), resulting in fewer opportunities to marry, particularly for women because of being perceived as asexual or not capable of being mothers (Rohwerder 2020).

Moreover, the literature highlights that stigma affects lived experiences in communities and within work places and healthcare, indicating that in addition to the social level of stigma, there is also a lack of institutional and legal support to enable inclusive participation.

Acquiring, keeping and terminating a job and remunerative employment were cited most frequently within major life areas (p8). Experiencing employment restrictions were found to result from misconceptions about persons with disabilities’ nature and ability. While compromised abilities were attributed to persons with disabilities independent of the type of impairment (Abah 2017; Habtamu et al. 2018; Mfaofo-M’Carthy & Grishow 2017; Mostert 2016; Rohwerder 2019a, 2019b), persons with mental disabilities experienced additional barriers to employment because of employers’ perception of them as ‘dangerous’, ‘lunatic’ or ‘unpredictable’ (Spittel et al. 2019; Stangl et al. 2019; Surur et al. 2017). Moreover, the literature reflects on employers’ fear that employing persons with disabilities would negatively affect their business (Abah 2017).

The literature reviewed strongly suggested that women with disabilities experience more challenges in receiving a job or being accepted as job candidates (Rohwerder 2020), which is commonly referred to as double discrimination because of intersectionality arising from gender and disability (Van der Heijden 2019; Van der Heijden, Abrahams & Harries 2019a; Van der Heijden, Harries & Abrahams 2019b).

The last chapter, community, social and civic life (p9), received more counts, distributed among participation levels than the other chapters. Community life, human rights, and political life and citizenship were most frequently reported. According to the literature, persons with disabilities’ participation in community life is characterised by social exclusion because of social stigma or self-isolation resulting from low self-esteem as a consequence of internalised disability stigma. Human rights violations against persons with disabilities are a result of different factors, including their lacking protection within the legal system, (Mfaofo-M’Carthy & Grishow 2017; Mostert 2016; Stangl et al. 2019; the Advocates for Human Rights 2016; UN CRPD Committee 2016), their fragility and vulnerability as a consequence of social exclusion, negative attitudes as enforcement of violations against persons with disabilities, misbeliefs about ostensible curing methods and being an ‘easier target’ given functional difficulties (Amoah 2016; Arulanantham 2014; Lord & Stein 2013; Mostert 2016; Rohwerder 2018, 2019a, 2019b, 2020; UNDESA 2016). Furthermore, persons with disabilities are often denied political participation, which limits their opportunities to claim their rights (Mfaofo-M’Carthy & Grishow 2017; Mostert 2016; Stangl et al. 2019; the Advocates for Human Rights 2016; UN CRPD Committee 2016).

## Insights from the data

This section shares further insights into participation restrictions related to community, social and civic life (chapter 9) that emerged through the interviews. Furthermore, it introduces a proxy for social stigma to explore its influence on participation in society.

### Participation restrictions related to community, social and civic life

During the interviews, five key themes emerged relating to this chapter: persons with disabilities’ willingness to participate, inaccessibility of community meetings, power differentials within community meetings, disrespect of persons with disabilities and the recognition of DPOs for empowerment. The survey included two questions examining the extent to which persons with disabilities in the region participated in DPO activities, and levels of participation in decision-making at community level (kebele^1^ meetings).

The interview data suggested that there is a strong willingness on behalf of persons with disabilities to participate in community meetings, social and civic life. However, opportunities to participate are restricted by specific disability-related access factors that posed a tension between willingness and realistic opportunity to participate. Irrespective of the type and severity of the impairment, seven types of barriers were reported: rurality and/or infrastructure; physical and/or architecture; legal and/or institutional; attitudinal and/or cultural; information and/or communication; socio-economic and/or material; and temporal. According to one participant:

‘There is a problem with accessibility […] advertisements are not written in braille; the clock rolls; the locations are uncomfortable; uncomfortable places, the inconvenience of transporting from the venue to the venue, the distance from the country.’ (P08^¶^)

For those who had an opportunity to participate, many shared that their voices were not heard or taken seriously. Four participants felt that they were either not listened to or silenced (P04, P05, P14^*^, P15^*^). A woman from Wereta stated that, ‘No, I don’t think I was involved, because when you sit down with others and your voice is not heard, there is a tendency to despair’ (P04). Feelings of invisibility were experienced by participants with differences in functioning, status, education and geographic location, but disproportionately by women (four women; one man). The way women with disabilities narrated their experience and their account of feelings of inferiority and invisibility suggests that gender norms intersected with disability norms. Except for two men from Debre Tabor (P01 and P06), all participants felt that their views were not taken into account. They frequently reported that their questions or comments received no (or undue) consideration. Participants pointed to the vicious cycle between underlying social norms, unequal participation and feelings of powerlessness:

‘It [*actions to making oneself heard by the community or government*] has no effect but speaking. The problem is not being accepted. We get frustrated and bored because of the lack of response. […] What we say is almost irrelevant.’ (P04)

According to the interview participants, this arises because of underlying embedded social beliefs about disability. Disability-related stigma that other persons with disabilities as ‘inferior, weak, and non-human creatures’ functioned as root cause of exclusion for our participants. As detailed by a woman, ‘In traditional mindset[s,] people think to close we [*meaning us*] blinded ones inside a house and [*we are*] not allowed to participate in public arenas’ (P05). Likewise, a man from the same district explained that ‘society doesn’t include you because it says that some people don’t have the means to go out’ (P08^¶^). Participant 14^*^ clearly expressed the community’s reservation against persons with disabilities’ participation ‘They don’t give us more information, so they don’t want us to participate there’ and Participant 13^*^ emphasised how her ‘hidden’ position in society constrained her participation ‘I have no recognition of any union and don’t have involved […] because I have spent my time by sit down at home.’ Another woman said that ‘a person with a disability cannot be considered doing anything in this district. We can’t go out in public with a disability; the people can’t see us’ (P03).

Disabled persons’ organisations were identified as a necessary bridge to overcome power differentials in kebele meetings, ‘the Disability Association is to achieve that we will have an influence when we are together and we will overcome problems’ (P11). Participant 08^§^ further reasoned that DPOs would enable persons with disabilities to be ‘organized and have a community to make a difference, so we are organized and getting a change’ and Participant 04 stated that ‘it’s a better way to go in union than one vote.’ As a result of patterns of exclusion, persons with disabilities emphasised DPOs as instrumental to accentuate their visibility and voice within the community:

‘[*W*]e are not able to reach the condition [*willingness of other community members to be represented by persons with disabilities*] without the activation of those organizations [*DPOs*] in rural and urban areas.’ (P05)

Yet, this study’s survey indicated that only 30% of participants are actively involved with local DPOs. Further interrogation of the survey data and follow-up interviews is recommended to explore the reasons why participation in DPO activity is so low among the participant communities.

### Assumed causes of disability

Following the widely accepted suggestion in the literature that disability stigma is particularly driven by misconceptions about the causes of disability, the following section explores how adults with disabilities understand the causes of disability. This provides insight into the ways in which disability stigma can be internalised by persons with disabilities and is likely to influence their self-esteem, value and self-worth.

Within the survey, adults with disabilities (*n* = 970) were asked to respond to the question: What do you think are the main causes of disability? Responses (response rate = 100%) were then classified into the following umbrella terms: evil spirit; curse of God; artificial and natural accidents; congenital and/or hereditary and/or natural forces; various diseases; lack of prenatal care; personal and environmental hygiene problem; psychological problems; I don’t know; other; multiple factors, such as evil spirit, disease etc. The study results suggest a knowledge gap about the causes of disability: 51% of the participants reported artificial and natural accidents, 18% mentioned multiple factors, 12% stated to be unaware and 8% believed in supernatural forces.

### Rights awareness among adults with disabilities

In exploring opportunities to claim rights, the survey sought to understand whether adults with disabilities felt aware and/or knowledgeable about their rights as persons with disabilities. Of the survey participants, 969 persons responded to the question ‘Do you know about your rights as a person with a disability?’ on a three-point scale (yes, partially and no). The results suggest that rights awareness is limited among the survey participants, with 52% indicating knowing about their rights, 23% reporting not knowing about their rights, and 25% reporting having only partial knowledge. Disaggregating the survey data on disability rights mirrored the patterns found for the survey data on assumed causes of disability.

## Discussion

While the literature generally supports the link between disability stigma and participation (Rohwerder 2019a, 2019b), a knowledge gap remains in understanding how these two experiences are linked. The present study’s findings suggest that embedded forms of disability stigma directly influence participation in at least three ways. Firstly, they can result in the full exclusion of persons with disabilities because of the inaccessibility of spaces and information. Secondly, when persons with disabilities engage in community meetings, they perceive that their contributions are belittled, not taken seriously, and thus they are prevented from participating in a meaningful way. Thirdly, and relatedly, the influence of disability stigma on efforts to participate can result in different kinds of epistemic injustice that arbitrarily reduce the influence our participants can have in the social practice of meaning making, with disability stigma undermining the status of persons with disabilities in their capacities as knowers.

### Disability stigma as full exclusion from social participation

Utilising the ICF framework to systematically untangle indications of disability stigma within the literature, the study findings help to explain how disability stigma limits participation opportunities at two levels: the personal and environmental. When information on meetings is shared in an inaccessible manner, or when community meetings are hosted in inaccessible places, persons with disabilities are fully excluded and unable to represent their own interests in such spaces (Table 4 and Table 5). The findings indicate that the labour of DPOs is critically important in connecting persons with disabilities with accessible information on community events, and in supporting and encouraging participation. All of the interview participants were active members of DPOs, but only 30% of survey participants were engaged in these collective disability-centred supports. This suggests that extending local DPO networks may be an important dimension of increasing participation in community meetings through raising awareness of opportunities to participate, and also providing tangible support to enable access. However, the study findings further suggest that raising awareness and providing support to facilitate access to meetings are not sufficient to address embedded socio-cultural norms and beliefs that prevent meaningful forms of participation.

### Disability stigma as restricted participation: The prevalence of testimonial injustice

Our findings from the literature and survey data point to specific forms of stigma that are unique to persons with disabilities and that influence social power dynamics during the process of participation. Firstly, our findings point to the persistence of social misconceptions around disability that ground disability stigma. In the literature, attitude (e4) was most frequently reported as an environmental barrier that was found to be driven by misconceptions about the causes of disability and a lack of awareness of what disability means. The interview data further helped us to reveal how those underlying stigmatising norms translate into lived experiences.

In-depth interviews with persons with disabilities about their experiences of participatory processes in the community pointed to ways in which disability stigma limits opportunities to meaningfully participate and therein manifests a case of testimonial injustice (Fricker 2007:1). In unfolding how interview participants perceived the dynamics between persons with disabilities as speaker and persons without disabilities as hearers during kebele meetings, the present study revealed examples of inferiority, disrespect and power imbalance: feelings of ‘not being listened to, not being heard, inferior, being silenced’. As speakers, persons with disabilities felt that hearers attributed less credibility to them than to their peers without disabilities. Central to their experience was the perception that being recognised as less credible was linked to their identity as a person with disability. Taking together with the results from the literature review and the survey data, the findings of this study support evidence of a ‘collective conception’ (Fricker 2007:15) grounded in the stigmatisation of persons with disabilities as unable to act as an authority with the necessary capacity to lead and contribute in a meaningful way. This study does not provide insight into the perceptions of persons without disabilities, rather it focused on how persons with disabilities narrated their experience of participation. The restrictions they experienced help us to understand how collective imagery of differences in power can result in unequal and unfair power relations and dynamics. In other words, the stigmatising norm acts as – what Fricker calls – ‘the prejudice that causes’ (Fricker 2007:1) a credibility deficit (Fricker 2007:17). While a deficit in credibility on its own does not necessarily result in a negative outcome for the subject, here, the cultural and social framing of persons with disabilities as less credible knowers manifest wide-ranging harm done to this group.

Secondly, the interview data […] findings also point to another aspect of testimonial injustice, an intersectional dimension. While feelings of neglect, disrespect and invisibility were shared by men and women with disabilities, women with disabilities appeared to be disproportionately affected. This aligns with Fricker’s (2007) framing of testimonial injustice within identity politics and points to the difficulties of single-axis and siloed approaches. From our insight into how disability stigma and gender norms interact, we suggest that inclusion-oriented policies must recognise persons in their full diversity. For the case of CBID, this requires contextual insight into the underlying gender norms and criteria that determine and legitimise one’s capacity as a knower as well as the drivers of those norms. This is necessary to address discriminatory norms and provide real equal opportunities for meaningful participation.

### Disability stigma as a driver of structural hermeneutical injustice

The distinct nature of disability stigma is premised on an assumption of differential power and knowledge between persons with and without disabilities. The survey found that almost 20% of persons with disabilities did not understand the causes of their disability or believed that it is because of supernatural forces. Moreover, only half of the participants recognised their basic human rights.

Through the ICF coding of the literature, it was found that participation restrictions were reported across all areas of life, providing evidence of the non-inclusivity of systems in society. In interviews, participants further pointed to the inaccessibility of meetings. Persons with disabilities expressed their concern about the absence of disability-inclusive communication and information, which would limit their opportunities to participate in society. Although it could be argued that those experiences are singular stories or accidental, this is countered by the scale and scope of our ICF analysis and survey data, which points to the systemic nature of disability stigma and exclusion. The survey data point to evidence that persons with disabilities are affected in their capacity as knowers with many internalising harmful beliefs and accepting a marginalised social position. Such marginalisation embeds acceptance of exclusion from processes of meaning-making and making sense of one’s experiences. As such, the authors suggest that disability stigma is not a case of accidental but structural hermeneutical injustice.

Taken together, the findings of this study suggest that negative norms in society translate into unequal knowledge and power systems that disproportionately affect persons with disabilities in their capacity as knowers. It is this epistemic difference that permits the structural injustice to occur.

## Conclusion

This study demonstrated that enabling the active participation of persons with disabilities in society remains a challenge for CBID, but active engagement with DPOs can be transformative for persons with disabilities in terms of social participation and knowledge and understanding of basic rights and entitlements. As showcased through the application of relevant ICF domains, there are many ways in which the participation of adults with disabilities in society is affected. However, restrictions regarding communal and interpersonal interactions appear to dominate. The fundamental point emerging from the reviewed literature is that stigma is a key hindrance to the participation of persons with disabilities in society, which is driven by underlying social norms and unequal power dynamics. In alignment, the analysis of primary data showed that misconceptions about the causes of disability persist and that underlying social norms related to gender and social identity also matter.

This study’s findings point to evidence of testimonial and structural hermeneutical injustice, but there is another dimension relevant to the case of disability. In both the literature and the survey results, it was found that processes of internalising stigma influence persons with disabilities in their capacity as knowers. Through developing key themes and subgroups of personal barriers, this article has shown that within literature, psychological processes of meaning-making were most frequently reported as personal barriers to participation, whereby internalised stigma and low self-esteem emerged as key concepts, directly linking the environment with the personal domain. This linkage received most attention for the last participation chapter community, social and civic life (p9), with a number of records reporting on self-isolating behaviour resulting from low self-esteem as a consequence of internalised disability stigma. This points to an additional dimension of epistemic injustice – what might be called ‘internalised injustice’ whereby harmful social norms and values are internalised by a person with disability and this can result in self-limiting behaviour. This comes *before* hermeneutical injustice. Further research is required on the extent to which internalising stigma results in self-limiting behaviour and whether this points to an additional dimension of epistemic injustice. However, this study’s survey findings point to initial evidence of how internalised stigma can result in self-limiting behaviour in three ways – the uncritical acceptance of misconceptions about disability by persons with disabilities themselves; limited awareness of one’s rights; and low membership in DPOs as institutions established by and for persons with disabilities. Furthermore, some of the interview participants described how harmful collective imaginaries are internalised and lead to frustration and/or dissociation from one’s agency. Further interrogation of the survey data to explore the dynamics of internalised stigma and how this generates epistemic exclusion as a form of internalised injustice is required.

## Footnotes

1 In the local language (Amharic), the word ‘kebele’ refers to the sub-district and/or community level. Subsequently, the expressions ‘kebele meetings’ and ‘community meetings’ are used interchangeably, describing meetings that are open to all community members, who live in the respective kebele.
